# Benefits in Cash or in Kind? A Community Consultation on Types of Benefits in Health Research on the Kenyan Coast

**DOI:** 10.1371/journal.pone.0127842

**Published:** 2015-05-26

**Authors:** Maureen Njue, Sassy Molyneux, Francis Kombe, Salim Mwalukore, Dorcas Kamuya, Vicki Marsh

**Affiliations:** 1 Kenya Medical Research Institute (KEMRI)—Wellcome Trust Research Programme, PO Box 230–80108, Kilifi, Kenya; 2 Centre for Tropical Medicine, Nuffield Department of Medicine Research Building, Oxford University, Old Road Campus, Headington, Oxford OX3 7FZ, United Kingdom; 3 Ethox Centre, Nuffield Department of Population Health, Oxford University, Old Road Campus, Headington, Oxford OX3 7LF, United Kingdom; University of Stirling, UNITED KINGDOM

## Abstract

**Background:**

Providing benefits and payments to participants in health research, either in cash or in kind, is a common but ethically controversial practice. While much literature has concentrated on appropriate levels of benefits or payments, this paper focuses on less well explored ethical issues around the nature of study benefits, drawing on views of community members living close to an international health research centre in Kenya.

**Methods:**

The consultation, including 90 residents purposively chosen to reflect diversity, used a two-stage deliberative process. Five half-day workshops were each followed by between two and four small group discussions, within a two week period (total 16 groups). During workshops and small groups, facilitators used participatory methods to share information, and promote reflection and debate on ethical issues around types of benefits, including cash, goods, medical and community benefits. Data from workshop and field notes, and voice recordings of small group discussions, were managed using Nvivo 10 and analysed using a Framework Analysis approach.

**Findings and Conclusions:**

The methods generated in-depth discussion with high levels of engagement. Particularly for the most-poor, under-compensation of time in research carries risks of serious harm. Cash payments may best support compensation of costs experienced; while highly valued, goods and medical benefits may be more appropriate as an ‘appreciation’ or incentive for participation. Community benefits were seen as important in supporting but not replacing individual-level benefits, and in building trust in researcher-community relations. Cash payments were seen to have higher risks of undue inducement, commercialising relationships and generating family conflicts than other benefits, particularly where payments are high. Researchers should consider and account for burdens families may experience when children are involved in research. Careful context-specific research planning and skilled and consistent communication about study benefits and payments are important, including in mitigating potential negative effects.

## Background

The provision of benefits and payments to participants in biomedical health research, in cash or in kind, is a common but often ethically fraught practice [1–3]. Much of the literature highlights challenges in determining appropriate *levels* of benefits or payments in different contexts, given the potential for these to become either an undue inducement or exploitative [2, 4, 5]. Ethical implications of the *nature* of a study benefit are much less commonly discussed and provide the main focus for this paper, with an emphasis on international research conducted in low-to-middle income country (LMIC) settings.

This paper addresses ethical issues around study benefits *and* payments, reflecting theoretical and practical challenges in distinguishing what should be considered a benefit or a payment in biomedical research. King (2000) describes three types of research benefit including: *direct* individual benefits arising from study interventions; collateral/*indirect* benefits to individuals, for example, free medical care including consultation, diagnostic testing and drugs; and *aspirational* benefits from the outcomes of research and their societal impact. For research payments, Wendler (2002) gives four categories, including *incentives* to encourage research participation; *reimbursement* to compensate for direct research-related costs such as fares for travelling; *appreciation* payments to show gratitude; and *compensation* payments to account for time and inconvenience during research. While these categories suggest that payments may be more specific in nature than ‘benefits’ in general, there are clear overlaps. Payments can be given ‘in kind’ rather than as cash, for example, goods given as an appreciation ‘payment’. Similarly, some benefits might be given in cash, for example where study participants are given cash to access local government health services (transport and other fees) instead of these services being provided by a research team during a study.

In the literature on the ethical issues around types of benefits given during health research, cash payments attract most attention and controversy related to an increased potential for undue inducement, clouding of participants’ judgment, and commercialization of the relationship between researchers and participants [3, 5, 6]. Also described in literature is the wide range of types of study benefits that are provided in research including cash payments, hard goods, foodstuffs, donations to local institutions, provision of stationery and medical services [7, 8]. For adult HIV studies, these have included providing legal services where participants are charged with minor non-criminal offences [8]. The provision of job opportunities to local community members has been seen as a significant community benefit in some setting [8], The provision of a broad range of benefits—including to the communities in which research is being undertaken—is in keeping with arguments for ‘fair benefits’ in international research, in recognition of underlying global structural inequities between those funding and conducting research, and participating communities [9]. Across this literature, there is limited information, particularly from low income settings, on community stakeholders’ experiences of study benefits and payments in practice, or their views on how these should be planned [8].

This paper draws on a qualitative study set up as a community consultation on study benefits and payments to support the development of locally responsive policies at an international health research programme in Kilifi, Kenya, and to contribute to wider international debates. Findings on the ethical work of boundaries for *levels* of benefits and payments from this consultation have been published elsewhere [10]. An earlier study explored common practices and the views of diverse research staff on study benefits and payments at the programme [8]. From these earlier reports, and as important background to the current paper, research staff and community stakeholders in Kilifi described risks in giving ‘too few’ and ‘too many’ benefits or payments. There was particular emphasis on potentially serious harms in giving ‘too few’ benefits in settings where many families are dependent on subsistence forms of livelihood, including farming, piece-meal labour, and casual and domestic work. These livelihoods present challenges for identifying appropriate levels of compensation for time, with any underestimates potentially impacting on a bread winner’s ability to feed their family on a daily basis. Risks of ‘overcompensation’ were also seen, primarily in relation to the researcher-participant/community relationships, including commercialisation, loss of trust and generation of rumours; but also in terms of benefits introducing inequities and disrupting values within families and communities [10]. As noted above, this paper focuses on the role of the *nature* of benefits in influencing these and other ethical challenges.

## Methods

### Study site

The study was conducted at the Kenya Medical Research Institute (KEMRI)-Wellcome Trust research programme (KWTP) in Kenya. The programme’s main centre is located in Kilifi County Hospital on the coast of Kenya, where a long term collaboration has been established with Ministry of Health managers and providers. Through this partnership, KWTP supports clinical services in Kilifi hospital and some peripheral clinics, including supplementing staff, supplies and equipment and providing a paediatric high dependency unit at the hospital [8]. Kilifi County’s population includes rural and semi-urban populations with the majority of the residents being from the Mijikenda ethnic group in Kenya. Statistics for the county show amongst the highest poverty levels, lowest literacy rates and highest indicators of gender inequity nationally [11, 12]. Amongst its activities, KWTP runs a Health and Demographic Surveillance System in the population of 260,000 people living within the hospital’s catchment area, in collaboration with County Health managers [13]. This is the population referred to as ‘the community’ in this paper.

Within the community, KWTP’s role in supporting clinical services is generally highly appreciated. It’s research role is less well and widely understood, contributing at times to concerns and rumours about common research practices such as taking blood and other biological samples, and the involvement of people who are well in research activities [12]. Given its close relationship with the community, KWTP has an active community engagement strategy to build mutual understanding and strengthen community input into research practice and policy [14]. Of relevance to this paper, these activities include regular meetings for information sharing and consultation with a network of around 200 voluntary KEMRI community representatives (KCRs), ‘typical’ community members selected by residents within smaller administrative geographic locations at public meetings to serve for a period of three years [15]. A team of community facilitators drawn from the community implement engagement activities; and other front line staff in the programme who support research activities (field workers) are also long term residents.

### Study population, sampling and data collection

A total of 90 research stakeholders participated, including i) 33 KWTP staff (community facilitators and field workers), ii) nine area administrative leaders (assistant chiefs), iii) 22 opinion leaders (leaders or members of community-based organizations, including women groups, youth groups and village dispensary committees), iv) 22 KCRs and v) four mothers of current study participants. These types of participants were chosen to represent a range of perspectives on research activities while ensuring some level of familiarity with research to facilitate debate and discussion. Within groups, participants were purposively selected to maximize diversity in gender, location (urban and rural), religion, age and education. Staff participants had a high understanding of research, including research ethics, from routine in-house trainings. All non-staff participants had had some prior contact with the programme, through their roles as KCRs, as guardians of study participants, through regular meetings with research staff (assistant chiefs) or through attending a KWTP open day (opinion leaders). A summary of participant characteristics is included in Table 1.

**Table 1:** Summary information for participants.

*Role*	*Total number*	*Gender M*:*F*	*Education range (years)*	*Religion*
Staff: Community facilitators	8	4:4	12-16y	Christian 6; Muslim 2
Staff: Field workers	25	17:8	8-16y	Christian 25
Assistant chiefs	9	5:4	12 y	Christian 7; Muslim 2
KEMRI Community Representatives	22	10:12	1-16y: 1-8y (14); 8-12y (7); College (1)	Christian 15; Muslim 6; Traditional 1
Community based organisation leaders	22	14:8	0-16y: 0-8y (11); 8-12y (8); college (3)	Christian 16; Muslim 6
Mothers of study children (malaria immunology study)	4	0:4	0-8y (3); 12y (1)	Christian 3; Traditional 1

**Fig 1:**
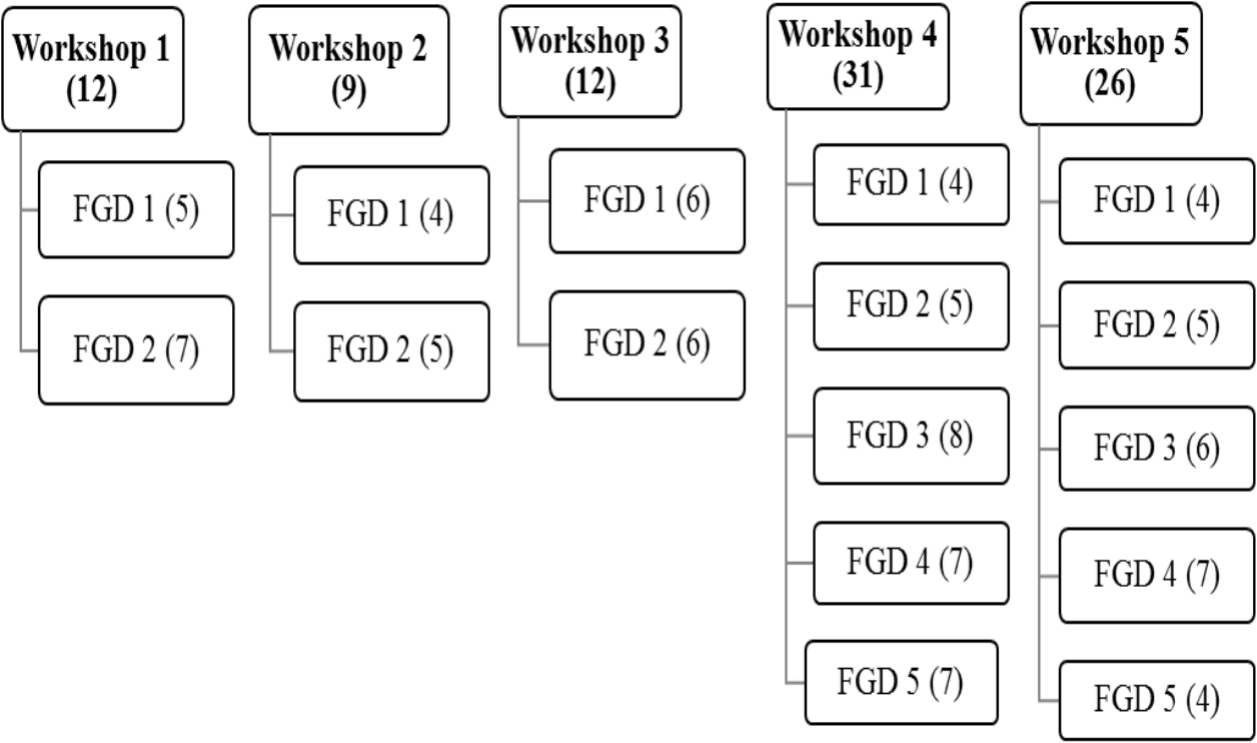
Flow chart representation of the two-stage consultation process (participant numbers in brackets).

The structure of the consultation included a half day workshop followed by a set of approximately four hour small group discussions within a two week period. Five workshops were held, each including between nine and 31 participants from one of the main groups described. After each workshop, participants were split into two to five small groups (see Fig 1). Workshops acted as a platform to share basic information about research, KWTP and ethical review processes, to introduce two hypothetical scenarios based on current practices for benefits and payments at KWTP and to begin discussions about these. The research scenarios, a home-based interview on health-seeking behaviour for childhood fevers and a facility-based Malaria Vaccine Trial (See Table 2), were used to support detailed discussion of study benefits and payments for very different types of research throughout the consultation. The small groups allowed for more in-depth, individualised exploration of issues and time for reflection on new concepts introduced in the workshop. During discussions, medical benefits, cash and ‘in kind’ payments and community benefits were particularly explored, reflecting types of benefits in common use at the KWTP. Facilitation encouraged debate within groups, including through cross-checking opinions across groups and the use of non-judgmental probes around emerging ethical issues. Discussions were held at venues convenient to participants in the language of choice (English, Kiswahili or local language). Details of the sampling and data collection methods have been given in greater detail elsewhere [10].

**Table 2:** Study Scenarios.

**Scenario 1: A home-based interview on fever treatment-seeking behaviour**
In a creative way tell a story about a mother who is being requested to participate in a research study that involves a one hour interview on her beliefs about the causes of fever in children. The story is aimed to bring out the information that would be considered in deciding whether to take part or not. *The main points that should be included in the story*: One hour one-off interview; mother can choose a convenient time for interview; study is about parent’s beliefs on causes and treatments for fever in children.
**Scenario 2: A mother is asked to allow her child to participate in a malaria vaccine trial**
In a creative way tell a story about a mother who is being requested to allow her young child to participate in a Malaria Vaccine trial. The story is aimed to bring out the information that would be considered in deciding whether to take part or not. *The main points that should be included in the story*: a Malaria Vaccine study, which is not a routine vaccine for children; has been shown to be safe in earlier studies and likely to be effective; A blood sample will be taken to test the health of the child before the vaccine is administered; the child will receive a health check after the vaccine is administered; follow up at home for 6 months by a KEMRI Field worker, 6 visits in total; in case the child is sick they will receive free treatment; when needed by the study team, transport will be reimbursed. Clinician is assigned to the dispensary to attend to all the sick children and other community members as a way of giving back to the community.

### Data management and analysis

At workshops, a note-taker made detailed notes of discussions. Small group discussions were audio-recorded, transcribed and translated into English by experienced staff fluent in local languages who had been present at discussions. Facilitators kept notes to capture the flow and dynamics of discussions. Following workshops and small group discussions, facilitators held debrief meetings to cross check perceptions and discuss emerging issues. Data were managed using NVIVO 10 and Microsoft Word applications, using coded identities. Data were analysed using a modified framework approach [16], involving in-depth reading of transcripts, making detailed summaries of discussions, developing a coding framework from major themes in the question guide and emerging issues in the transcripts, and coding data across all transcripts. Two set of analysis charts were developed to compare information across and within groups; and to chart changes of opinion and dynamics across debates. Analysis was primarily conducted by VM and MN with support from other authors, using an iterative process. The main inductive themes were perceived challenges for ‘too many’ and ‘too few’ benefits, the role of time and study procedures in perceptions of appropriate benefits and the implications of different types of benefit. Interpretation drew on assessments of the strength, nature and level of support for opinions within the consultation overall, and the literature on benefits and payments and research ethics in general.

### Ethical review

This study was reviewed and approved by the Kenya Medical Research Institute (KEMRI) Scientific Steering committee and the Kenya Medical Research Institute (KEMRI) Ethical Review committee. Verbal consent was sought from workshop attendees, documented in minutes. Written consent was given for participation and audio-recording of discussions in the small groups. The use of both verbal and written consent procedures for this study were reviewed and approved by the Kenya Medical Research Institute (KEMRI) Scientific Steering committee and the Kenya Medical Research Institute (KEMRI) Ethical Review committee.

## Findings

The findings are presented in sections reflecting views on different types of benefits, including the relative roles of cash and goods as benefits, medical services and wider community benefits.

### Provision of cash versus goods

There were mixed views on the relative appropriateness of cash and non-cash benefits, with the advantages and disadvantages of these often working as mirror images of each other.

#### Views about cash payments

The main advantage of giving cash benefits, raised repeatedly by staff and community stakeholders, was the flexibility this would allow participants to prioritise their own needs. Researchers might otherwise identify benefits that were not particularly useful:

*… Its better the money* (laughs)… *you are the bread earner*, *you come here and waste your time and then the child is given a ball*. *Fine she will play with the ball but what will they eat to have energy to play with the ball*? *(P1F12*: *Study mother*, *40yrs*, *12yrs education)*

For child study participants, mothers would also be able to share cash benefits amongst siblings, often seen as preferable to providing only for the child involved in a study, particularly for the most-poor where this might be used to buy food.

The main issues for cash payments were linked to a potential to heighten all the risks associated with giving ‘too many’ benefits, described in detail elsewhere [10]. These risks included generating family conflicts where mothers of children participants in this traditionally patrilineal community are given cash payments, since men are generally seen to control household resources; and increasing risks that people will take decisions without sufficient understanding or reflection on the implications, described as problematic in undermining people’s rights to free choice and creating practical difficulties where participants join studies without fully understanding what will be required of them. Cash was also seen as potentially problematic in changing relationships between researchers and study participants towards an increasingly business-like model, particularly seen as undesirable where children participate in research (where there’s a risk of research participation becoming ‘like selling children’) or where cash is given ‘in exchange’ for uncomfortable procedures such as blood taking, which would be seen as ‘buying blood’:

*When it comes to money*, *people have different perceptions*. *They might not see it as a gift*. *The material benefits can be the same in quantity to the money*, *but because of the different nature of people*, *their perceptions and conditions are different*. *Some may create doubts because of money*. *(P7F15*: *Village Dispensary committee member*, *Male 42 y*, *8y Education)*

Cash specific issues were also described. These included the evident face value of cash benefits, making it critical for researchers to set the ‘right’ value for this form of benefit, while goods were seen as less readily interpreted in terms of their cash value. In addition to the risks above that cash benefits could act as too great an incentive to participate in research, there were concerns that giving very small amounts of cash would be perceived as insulting. The value of cash to different families was also seen as complex since amounts of cash seen as small to some families might be highly significant for the most-poor families.

*Money there’s no little money*, *yeah fifty shillings can be … a lot of money to somebody*. *(P6F5*: *Field worker*, *Male*, *41y)*

There were concern that cash benefits could be quickly and easily compared with earlier and others’ experiences of these, potentially creating dissatisfaction where the reasons for different values (for example, more time spent or inconveniences incurred) were not obvious. These views about the challenges of setting cash values on benefits were reflected in these consultations themselves, with many finding it difficult to give recommendations on what the WTRP guidelines should specify:

*This question will … remain unanswered*. *It seems to be a simple question but it’s a tough one*. *It’s not easy to give a figure which will be reasonable to everyone*. *(P2F12*: *Mother of study child*, *8y education)*

Further practical issues described included greater security of risks for staff carrying cash and the potential for cash to be mismanaged by staff.

Specifically, cash payments for studies conducted at home (for example, in compensation for time) were thought to be an unfamiliar practice and likely to raise concerns and questions. Most people would more commonly associate cash payments with reimbursement of travel costs, for example, to attend a study clinic. Even this would not necessarily be straightforward. A field worker described complications for a research clinic where payments were made in the guise of transport refunds, although in reality this was to compensate for time and inconvenience in walking to a study clinic since no public transport was available:

*You know X has no public transport…and the mothers who used to participate in those studies …used to be given fares*. *So they were asking*, *“Why are you giving me fare refund*? *There are no matatus (minibuses)*, *there are no bodabodas (motorbike taxis)*?*” (All laughing)*. *So it became difficult to answer*! *(P5F4*: *Field worker*, *male*, *36y)*

#### Views about goods as benefits

Given the issues raised for cash payments, some community members felt that goods such as food stuffs were a better alternative, particularly for research conducted in the home. Goods were seen as more familiar, for example, being common as a social norm for visitors to the home, and as a typical benefit-sharing practice for non-governmental organisations working in homes in the area. One suggestion for making goods more acceptable as a research-related benefit was by providing items closely related to the nature of the research, including educational materials (for example, leaflets) or other resources for the health issues addressed (for example, water purification tablets or hand-washing soap for diarrhoeal disease research). Educational benefits were described by one community stakeholder as having the potential to outlive those of cash.

*…You can compare money to water; once it’s poured that’s the end of it*. *Even if you were to come the following day you would not be able to identify where you had poured the water*. *But when it is something material for example it will last longer and they can keep that for a long time 5*, *6 years remembering that KEMRI once visited them… (P2F15*: *Muslim Leader*, *Male*, *68y*, *8y education)*

Specific practical challenges for goods, in comparison to cash, were difficulty in carrying these around large study areas; and risks of generating jealousy between neighbours, given their high visibility (whereas in Kenya cash can easily be sent through a mobile phone transaction). Just like cash, goods were seen to carry risks of generating negative rumours about researchers’ motivations:

*…you might bring [a bag of] flour and …unfortunately I start having diarrhoea*. *I will start saying*, *it’s the flour that came from KEMRI that…has made people have diarrhoea*. *(P3F7*: *Rural*, *Assistant chief*, *female)*

Overall, a key point about the provision of goods was that this was mostly viewed as better than cash when giving ‘tokens of appreciation’ to research participants. For compensation for direct costs incurred there was a strong preference for cash as a mode of payment, and this was also often true for compensation for amounts of time likely to impact on income earning potential that day:

*Food [given during clinic visits] is not enough… these mothers tend to spend most of their hours there*, *waiting for treatment and then they are taken back home…their time is not compensated*, *it’s just food they are being given*. *(P5F4*: *Field worker*, *male*, *36y)*

### Provision of medical services

#### The importance of research-related medical benefits

Medical benefits were mainly discussed as part of the Malaria Vaccine Trial scenario (MVT), where medical services featured prominently (see Table 2). Appreciation of KWTP medical services were linked to perceptions of high quality, convenience, speed and consistency of access in comparison to government health services, although the latter are officially free to children under the age of years in Kenya. These attractions of research-related medical services were seen as particularly relevant for those who could not afford the alternative of paying for private health care.

*… I’d want to talk about poverty*, *because you find that majority of those who join research are those who can’t afford treatment*, *most of them*. *So when they are enrolled or they are consenting*, *they immediately think about the treatment part… as opposed to some who can afford going to private clinic X …(P6F2*: *Fieldworker*, *male)*

Since the medical services provided by researchers in the MVT scenario were often closely related to the nature of the research, most participants felt they were appropriate, and less likely to be misinterpreted or cause rumours than cash or ‘in kind’ payments. Many felt it was still important for the child to benefit directly given their experience of discomfort from samples being taken in this study. Since—as before—cash benefits would risk interpretation as ‘selling blood’, strong suggestions were made by many staff that research samples should be used to provide health checks for child participants, and treatment offered where needed. An example was testing for and treating anaemia, where found.

Where substantial amounts of time were used in research participation, medical services—however valued—were not regarded as adequate compensation [10]. Viewed in the context of common patterns of underlying poverty, time was related to a breadwinner’s capacity to feed their family at the end of each day. Cash would therefore be needed to compensate for significant amounts of time, where these were incurred, in addition to any medical benefits provided. Medical services were also viewed as an uncertain form of benefit, since individuals would only benefit if they became unwell. For this reason, some participants saw medical benefits as a ‘mixed blessing’:

*A disease is something that comes naturally*, *so there is no person who has prayed for her child to wake up the next day sick so she can be in the study [get medical benefits from the study]*, *no there isn’t*. *(P3F7*: *Rural Assistant Chief*, *Female)*

The existence of free medical care to children under five in government health services also undermined the idea of research-related medical services as a true ‘benefit’:

*I don’t think any study now can talk about free treatment as a benefit… all children under five receive free treatment from the government … Maybe we say they will be attended to immediately…then that would qualify it as a benefit*. *(P3F1*: *Community facilitator*, *male)*

Other instances where medical services were not particularly valued included where participants had health insurance or could pay for private health care; or were adults, considered to need medical care much less frequently.

#### Extension of medical services to the family

Across studies at KWTP including research-related medical services as a benefit, there are variations in the nature of services included and who has access [8]. For research involving young children, questions were raised about who else within the family should have access to research medical services and under which circumstances. Particular candidates for additional access were mothers and child siblings; a practice for some long-term longitudinal cohort studies in KWTP. Views that siblings should be given access to research medical services were based on an argument that mothers would be additionally inconvenienced if obliged to seek care for an unwell sibling and study child in different places. A similar argument applied to unwell mothers accompanying a study child to clinic, who would also have to seek care in two places. In these situations, an older child might often be taken out of school to assist the mother:

*…maybe the sibling to the study participant is also sick … the mother might stop one of their siblings going to school to assist*. *[Or]… the inconvenience for the mother queuing for the study participant… [then] moving to the other bench queuing for that kid [sibling] (P4F1*: *Community facilitator*, *Male)*

In some studies where children were followed up at home over time (for example, longitudinal malaria immunology cohort studies), field workers monitor study children’s health, including screening and testing fevers for malaria, or expediting referral to nearby health centres for other illnesses. Staff participants described discomfort where a sibling of a study child was unwell at the time of their visit, but they should not—under study protocol—offer the same services, even if the children appeared to be suffering from the same condition. Field workers and reportedly mothers saw this response as unfair, generating challenges in attending research clinics (as above) and potentially in withdrawing from study on the basis of this perceived unfairness.

*… (Continues) Because if we say we have to turn away the mother or tell the mother go and queue to the KDH clinician… I don’t think if she’ll participate in any study again*. *(P4F3*: *Field worker*, *female*, *32y)*

Many staff felt that researchers should provide care to an accompanying mother or young sibling unwell on the day of clinic attendance for a study child. However, the potential for these additional family services to act as an undue influence on parents’ decisions to participate were raised within these discussions [10], and remained an issue for some community members. Other potential challenges to expanding medical services in research were negatives impact on quality and timeliness of those services, on research staff morale, and on the relationship between research and government health workers where the latter felt their roles were being undermined. To limit some of these challenges, compromise suggestions including providing consultation services free of charge but not subsiding costs of treatment. While adults were seen as likely to experience fewer illness episodes than young children, perceptions of higher costs in of treating adult illnesses underpinned views that studies involving adults should not extend medical benefits to their families.

### Community-wide benefits

The main form of community-wide benefit currently provided by KWTP is strategic support to routine government health services in the county through a close collaboration with the Ministry of Health in Kilifi. As described in the introduction, these community-wide medical benefits include extra staff, supplies, equipment and infrastructure, and aim to ensure that good medical care is widely available regardless of involvement in research [8]. Most of these services are provided long term, but additional community-wide medical services are included in many studies during the lifetime of those studies. During our discussions, we provided information on community-wide medical services because their integration into routine care makes them relatively invisible to many in the community.

Community-wide medical benefits were viewed as an important way of ‘giving back’ to the community in return for their support to research, building and cementing the relationship between the community and the research organisation. At times, this was seen as a responsibility for researchers:

*Even though research is voluntary*, *we should remember that this research will benefit the community and KEMRI staff…without the community*, *no work will be happening*. *So why shouldn’t you give benefits to people participating in your research*? *(P3F9*: *KCR Member*, *Male*, *55y*, *12y education)*

In previous consultations with staff at KWTP, community-wide benefits had been particularly strongly promoted as a means of providing extra medical support in a largely low-income community without risking individual forms of undue inducement (Molyneux 2012). This was seen as a way in which relatively highly resourced researchers could respond ethically to global structural inequities reflected in low access to good health care in settings such as Kilifi. These views were underlined in some cases, but not all, by perceptions of the relative wealth of the research institution. These views were echoed by some in this consultation, with community-wide medical benefits seen as fair compensation in some instances, given the challenges described for cash and goods, but this was limited to situations where inconvenience was relatively minor, for example, short periods of time spent in an interview. Importantly, community-wide benefits—of any type—were not seen as reasonable individual compensation for longer periods of time, given the economic implications of time in this community. While study participants might often have sacrificed important economic activities, they would gain the same benefits as non-participants. This was seen as unfair and as a disincentive to participate in research:

*…within that 1 hour [of research participation] you could have done something big…and the research you will be doing will not benefit this mother alone but many people*. *So the other many people will be using that 1 hour doing their own duties and then they benefit from that research*. *So I think the mother should be given something… (P1F13*: *Village Dispensary Committee Member*, *Male 58yrs*, *12yrs education)*

### Information and communication as cross cutting issues

Across discussions, a frequently emerging issue was the importance of information and communication about study benefits, where the latter was seen as a centrally important feature of good practice.

#### Information as a benefit

Across groups, participants frequently commented that learning more about health, including particular diseases or conditions under study, was an important benefit for individuals. Whether specific efforts were made to provide education, including educational materials, or study participants gained knowledge informally through their interactions with research staff, these were seen as valuable. Several participants underlined this value for study participants who were economically stable, where learning gained through participation might be sufficient, and would—as noted earlier—likely outlast many other forms of benefit.

*I would say that benefits do not necessarily have to be money*, *it depends on the particular research participant… other participants do not even need money or flour*, *what she will learn is enough for her*. *(P4F14*: *Community Development Fund Chair*, *Female*, *43y)*

#### The critical role of communication

Effective and respectful interpersonal communication were seen to be an important influence on good practice in relation to study benefits, alongside other factors such as minimising inconvenience, for example, by allowing choice in timing of activities, or by careful planning of studies [8]. Importantly, the ability of research staff to communicate well with study participants underpinned understanding and acceptability of research, as well as the way any benefits would be perceived:

*The KEMRI officer will not just give out something… and walk away*. *There is a discussion…like ‘I’m grateful*, *you welcomed me well*, *thank you for your time and I think it’s good as I leave I give you something small that will help you and your children’… I don’t think if this person had that explanation… she will come to think that I have sold my service to KEMRI or I have been given this because blood was taken…’ (P1F7*: *Urban Assistant Chief*, *Female)*

As a specific communication issue in this wide geographic community where different studies are often conducted simultaneously as well as over time, it was described as essential to give clear explanations on why benefits might vary between studies.

## Discussion

This study set out to explore the experiences and informed views of community stakeholders in an international research programme in rural Kenya on good practice in providing different *types of study benefits* to participants. Based on practices common in KWTP and other similar settings, the main types of study benefits explored were cash, goods and medical services for individuals, and community-wide benefits. In this discussion, we focus on key findings in these areas and, drawing on their relationship to the literature, propose components of good practice for planning study benefits in this and similar settings. A detailed description of findings from this consultation on the influence of *levels of study benefits* (their financial or economic value) has been published separately [10]. Recognising that ethical issues around levels and types of study benefits cannot be clearly separated, we explore this interaction within the discussion.

Across all types of benefits discussed in this consultation, an important emerging theme was the importance of skilled, consistent and respectful inter-personal communication with study participants about why particular benefits were being offered, and why these might vary between studies and over time. Communication also provides an important opportunity to show appreciation directly, with or without any accompanying material benefits. Similarly, health information was often highly valued as a long term benefit. In many situations, we note that providing consistent communication about benefits across studies and over time to participants and communities will be important, and depend upon there being an agreed and reasonable standardisation of approaches towards study benefits within research institutions. For the same reason, the budgetary and logistical implications of study benefits should be considered early in the planning process.

The methods used in this study were designed to allow in-depth informed discussion and debate around dilemmas in benefit sharing that often draw on concepts in the ethics literature not necessarily intuitively understood by many community stakeholders, such as ‘undue inducement’ [17]. At the same time, the methods aimed to avoid privileging ethical issues in the literature at the expense of those identified by community stakeholders. These methods, based on a deliberative participatory approach, have potential methodological challenges, particularly in ensuring balanced representation of different positions [18, 19]. Examples are that facilitators’ attitudes and group dynamics may have influenced the views expressed. At the same time, information sharing and group debate have been centrally important in this study in mapping a range of informed opinions to feed into a policy-oriented analysis. Facilitators have aimed throughout the study to maintain awareness of positionality [16], for example through encouraging open discussion and using careful and non-judgmental probes around ethical issues. Ironically, in this study on study benefits, one such potential influence was that KCRs were reimbursed for their time and travel costs for participation in the study itself. Nevertheless, any effect of these payments may be limited by their routine use across KWTP community engagement activities involving KCRs.

### Dilemma of providing cash or goods

In the literature, cash benefits have been argued as important in maximising choice for participants [20]. They have also been associated with increased risks of commercialization of the researcher-participant relationship [1, 2, 6], and—for research involving children—use in ways that do not benefit study participants [3]. Those who argue the importance of choice, as an autonomy issue, see removal of this option as an example of over-paternalism on behalf of researchers [2]. Arguments that cash payments should be avoided for the most-poor given increased risks of undue influence are also countered by recognition that withholding cash payments for this group would be unfairly discriminative [3].

Across this consultation, community stakeholders made similar arguments. Notably, in comparing cash and goods of the same value, cash payments were associated with an increased likelihood of many risks associated with ‘giving too many benefits’ [10]. The greater the financial value of the benefit, the more cash was seen to accentuate the risks involved. These included a higher risk of unacceptable degrees of influence on decisions to participate in research, and generating family conflicts and rumours within the community, particularly where cash payments in research were unfamiliar.

Amongst these risks, those of undue inducement and commercialisation, that is, that the relationship between researchers and participants would look like one based only on ‘buying and selling’, were particularly emphasised. These were also importantly countered by the importance of choice given by cash payments, *particularly for the most-poor in this community*. For research involving children, a strong fairness argument was made for the capacity of cash to allow mothers to share benefits between siblings, again prioritised for the most-poor. A challenge raised for cash for family dynamics was premised on gendered patterns seen as common within households in this traditionally patrilineal community; fathers ‘should’ traditionally have sole control over household resources, particularly cash. This view was controversial within these debates, on the basis of being unfairly discriminative towards mothers and unrepresentative of the community at large, particularly over more recent times. It also raises controversy in relation to wider principles of unfair discrimination.

A further challenge for cash payments was that the highly visible financial ‘face’ value of cash was seen as likely to prompt comparisons of benefits between studies and over time in ways that can create dissatisfaction with studies and the research programme. The actual amount to be paid as cash (unless for reimbursement of costs) was also seen as very hard to establish since appreciation would be closely influenced by levels of need. There would be a high risk that the same payment would act as an undue inducement in some families and seem insulting in others. Practical challenges for cash included security risks for staff carrying cash and risks of mismanagement by the same group.

Giving goods as study benefits was highly valued in this consultation, as long as these were useful. But goods were not devoid of the risks associated with cash, potentially working in exactly the same way to generate undue inducement, complaints related to comparisons made between studies and over time, rumours and family conflict. Further, and importantly, goods were seen as most appropriate as forms of *appreciation or motivation* for participating in studies, and less so as *compensation for time* where this has a significant economic value [10]. In this community, where poverty and subsistence forms of livelihood are relatively common, the economic value of time was associated with a capacity to earn enough money to feed families on a daily basis. Significant amounts of time taken in research should therefore be compensated with enough cash to buy food for the family that day, although some argued that equivalent amounts of foodstuffs (such as flour and dried beans) would be a reasonable alternative.

Comparing scenarios, a key influence on the risk of cash generating many of these problems was the site of the research interaction. Cash payments were seen as particularly sensitive if made during household visits, as compensation for extended periods of time (for example, lengthy interviews or group discussions), in which case goods were seen as more acceptable. Cash as compensation for time was less sensitive when given for attending research clinics outside the home, when transport costs were already being reimbursed. Transport reimbursement could clearly not be made using goods. To a large extent, these views reflected common understandings and expectations of study benefits. Cash payments are routinely made to reimburse travel costs, whereas compensation for time spent in research is a much less familiar concept. It seems that giving *extra cash* to compensate for time *in addition to reimbursing travel costs* is less visible than when time spent at home is compensated in this way. Skilled and consistent communication about the reasons for different forms of benefits and payments may lessen this risks, but giving cash payments at home should be seen—at least in this context—as potentially more sensitive.

### Medical services as a benefit

In previous research on benefits and payments in Kilifi, many researchers and health managers placed a high value on providing medical services to study participants as a form of study benefit [21]. Providing medical services was seen as a way of balancing the benefits and burdens of participation that would have a lower risk of generating undue inducement or commercialisation than other forms of study benefit. Since the provision of medical services is a core activity in health research, additional advantages were seen in drawing on existing resources and skills.

Across this consultation, stakeholders saw the medical services provided by KWTP within studies as an important benefit for participants, particularly for research involving children for whom the acute illnesses often managed in this way (for example, malaria or pneumonia) were recognised as more common. KWTP services were generally seen as being of higher quality, more reliable and quicker to access than government health services [8, 22], although the latter were officially provided free of charge to children under the age of five years. Given these advantages, research-related medical benefits were widely seen as likely to influence many positively in deciding to participate in research, with highest risks for the most-poor [23].

At the same time, medical benefits—like goods—were seen as mainly being appropriate as a form of incentive or appreciation for participants, and not as a compensation for time spent in research, where the latter was economically significant. In addition, several pointed out that medical services would only act as a benefit if and when children became ill. Not only was this uncertain, but it was a circumstance that could not be hoped for by parents. Thus, as noted above, the time spent in participating in research should be separately assessed in considering appropriate study benefits overall.

A particular issue for medical benefits explored in this consultation was the issue of *who within a family should have access to the services provided to young children participating in studies*. This question had been generated during earlier research, when field workers at KWTP described perceived unfairness during interactions with families in which mothers or siblings of study children were unwell, but protocols allowed for medical services to be provided to a study child only [8, 24]. The same issue was recognised in this consultation, framed as a harm in generating *additional* burdens for mothers if medical services were limited in this way. If a sibling of a study child was unwell at the time of a scheduled research clinic appointment, mothers would have to find a way of seeking care for the sick sibling separate to keeping the research appointment. If the study child was also unwell, the dilemma would be greater since an option to skip the research clinic appointment (highly inconvenient to researchers) would be removed. The same situation would occur if the mother was unwell at this time. One strategy would be to take an older child out of school to assist. In any case, the mother would be forced into a position of prioritising the care of one child over another, widely seen as unreasonable.

The view that the interests of a study child should not be placed above those of other children in the family—described here for medical benefits and, earlier, for other forms of study benefit—emphasises that the research approach of placing a high importance on study children as individuals, separate from their families, does not fit well with local conceptions of good practice, reflecting similar positions in the literature [25]. It has been strongly argued and widely accepted that a ‘fair benefits approach’ in research should consider wider communities as potential beneficiaries as well as individual study participants [26]. From our findings, it can also be argued that researchers should consider the position of a study child’s *family* in planning fair benefits. Involving children in research inevitably engages other members of the family. At the same time, mothers, who are generally (in this setting) responsible for taking care of children, must be equally concerned about the welfare of all their children. On this basis, researchers have a responsibility to ensure that their activities do not compromise the position of families they engage with. The suggestion made in this consultation that researchers should at least offer urgent medical services to siblings or caretakers who are unwell at the time of scheduled clinic visits, would fit with meeting this responsibility.

### Community wide benefits

The ‘fair benefits approach’ described earlier focuses on the importance of taking account of wider contextual issues that generate ethical challenges for researchers, rather than focusing narrowly on a balance of benefits and burdens to individual participants [26, 27]. By this account, a wide range of types of benefits and beneficiaries—including community-wide benefits—are important for researchers to consider. Particularly for international research in low-income settings, this argument identifies a need for researchers to act in ways that contribute to diminishing existing global inequities in access to health and other resources; community-wide benefits are one way in which this can be achieved. Community stakeholders in this consultation supported this view, seeing community-wide benefits as an important way of researchers’ ‘giving back’ to the community, acting as a form of appreciation and recognition of unmet health needs [8, 17]. This form of benefit was seen to build trust and good relations between researchers and the wider community. At the same time, community-wide benefits were not seen as a substitute for individual study benefits, including those needed to show appreciation, motivate or compensate participants for their time or for inconvenience. As described in the literature, individuals may not be willing to take up the risks and inconveniences of research participation for the sake of benefits that will be enjoyed by the wider community [27].

## Conclusion

The methods used in this study have generated in-depth, informed and reasoned discussion that can support planning for good practice in study benefits for participants, their families and the wider community. In research, the nature of what is given has an influence that is both separate from and related to the financial value of a study benefit. In comparison to study benefits given as same-value goods, cash is more likely to heighten risks associated with giving high levels of benefits, notably undue inducement and commercialisation of researcher-participant relationships. Higher values of benefits are likely to accentuate this difference. However, in addition to reimbursement of financial costs, cash reimbursement of economic costs of participation may be very important, particularly for communities where subsistence livelihoods are common. Cash also importantly strengthens choice and the likelihood that benefits are realised in practice. The importance of risks associated with benefits may be influenced by the context for research; for research institutions like KWTP, where researchers are reliant on strong long-term relationships with a given geographic community, risks of commercialisation from the use of cash benefits are particularly important.

As for all study benefits, risks associated with giving cash can be minimised by skilled and consistent communication about its purpose; avoiding unfamiliar practices, such as giving cash at home; and planning studies to limit inconvenience, including by maximising flexibility for participants. Goods and other forms of study benefits, including medical services and health information, provide good incentives for and appreciation of participation, but can generate negative effects without careful communication and are unlikely to compensate for significant economic costs. In planning study benefits for research involving children, researchers should take account of the study child’s family, recognising that benefits targeting this child alone may generate hidden burdens for their families, and therefore for the child. Interpersonal communication is key to maximising benefits and limiting burdens in research, including through showing respect and appreciation for participants’ contributions, and building understanding of the reasons benefits are offered in research, and why these may differ across studies and over time. Careful planning in advance, supported by locally responsive strategic approaches to benefit sharing, are important to support the implementation of good practice.
